# A Multi-D-Shaped Optical Fiber for Refractive Index Sensing

**DOI:** 10.3390/s100504794

**Published:** 2010-05-11

**Authors:** Chien-Hsing Chen, Tzu-Chein Tsao, Jaw-Luen Tang, Wei-Te Wu

**Affiliations:** 1 Department of Physics, National Chung Cheng University, Chia-Yi 621, Taiwan; 2 Department of Mechanical Engineering, National Chung Cheng University, Chia-Yi 621, Taiwan; 3 Department of Biomechatronics Engineering, National Pingtung University of Science and Technology, Pingtung 912, Taiwan

**Keywords:** multi-D-shaped fiber, femtosecond laser, sensor, refractive index

## Abstract

A novel class of multi-D-shaped optical fiber suited for refractive index measurements is presented. The multi-D-shaped optical fiber was constructed by forming several D-sections in a multimode optical fiber at localized regions with femtosecond laser pulses. The total number of D-shaped zones fabricated could range from three to seven. Each D-shaped zone covered a sensor volume of 100 μm depth, 250 μm width, and 1 mm length. The mean roughness of the core surface obtained by the AFM images was 231.7 nm, which is relatively smooth. Results of the tensile test indicated that the fibers have sufficient mechanical strength to resist damage from further processing. The multi-D-shaped optical fiber as a high sensitive refractive-index sensor to detect changes in the surrounding refractive index was studied. The results for different concentrations of sucrose solution show that a resolution of 1.27 × 10^−3^–3.13 × 10^−4^ RIU is achieved for refractive indices in the range of 1.333 to 1.403, suggesting that the multi-D-shaped fibers are attractive for chemical, biological, and biochemical sensing with aqueous solutions.

## 1. Introduction

Traditionally, the plastic cladding of the optical fiber was easily stripped by CO_2_ laser machining to expose the fiber core [1,2]. However, the core and cladding layers of communication grade multimode optical fiber are composed of fused silica, which is a transparent, hard, brittle, and high band gap (∼9 eV) material that could not have been effectively machined by long-pulsed lasers (e.g., CO_2_ laser, pulse duration at range of μs). The peak power intensity of the long-pulsed laser is not high enough to generate a significant amount of free electrons [3–5]. Recently, femtosecond laser has been extensively used for microfabrication. The most prominent features of the femtosecond laser over conventional long-pulsed laser are ultra short pulse duration and very strong peak power intensity, which can photoinduce the non-linear multi-photon absorption of a material during irradiation. The material vaporizes immediately after absorbing ultra high transient pulse energy from the ultra short pulse of femtosecond laser [6,7]. It can be used to engrave on transparent, hard and brittle materials very precisely, such as optical fibers, without inducing any micro cracks and heat affected zone [8,9].

**Figure 1.**
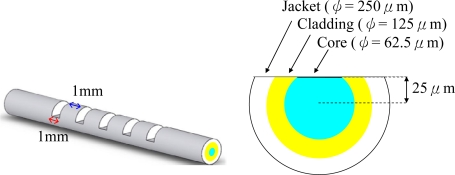
Illustration diagram of the multi-D-shaped optical fiber.

In this paper, we report a multi-D-shaped optical fiber sensor with a direct write technique by using high-power femtosecond laser pulses. A communication grade multimode optical fiber (Corning 62.5/125 optical fiber) was adopted in the present study. The optical fiber was composed of a silica-based core (62.5 μm in diameter), and cladding and polymeric jacket with outer diameters of 125 μm and 250 μm, respectively. Shown in Figure 1 is an illustration diagram of the fabricated multi-D-shaped optical fiber. For a single D-shaped zone the depth was 100 μm measured from the surface of the polymer jacket layer, and the length was 1 mm. The distance between two neighboring D-shaped zones was 1 mm. The number of D-shaped zones fabricated could range from three to seven, and they were distributed in line along the axis of the optical fiber. The operating principle of sensing is based on attenuated total internal reflection (ATR) via multiple internal reflections along the fiber and the attenuated light intensity of the multi-D-shaped fiber changes linearly with an increase of the surrounding refractive index. The loss of light energy caused by the sensing portion of the fiber is detected by a sensor interrogation system which is used to derive the refractive index of the surrounding media. In this scheme the fiber plays a role not only as a signal transmission line but also as a sensing component. The result of the tensile test of the multi-D-shaped fiber is reported. The ability of the multi-D-shape fiber as a high sensitive refractive-index sensor to detect changes in the surrounding refractive index was also studied.

## 2. Sensor Fabrication

**Figure 2.**
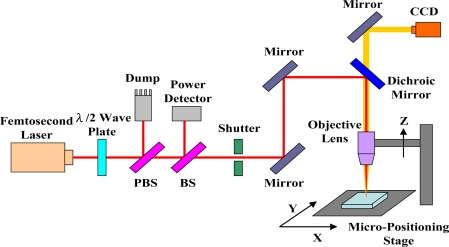
Experimental setup of a femtosecond laser micromachining system.

A femtosecond laser micromachining system, as illustrated in Figure 2, was used for engraving the trench on the optical fiber. The femtosecond laser was a regenerative amplified mode-locked Ti: sapphire laser with pulse duration of ∼120 fs after the compressor, central wavelength at 800 nm, repetition rate of 1 kHz, and maximum pulse energy of ∼3.5 mJ. The energy of the linear polarized Gaussian laser beam was adjusted by a rotatable half-wave plate and a polarizing beam splitter (PBS). A certain fraction of the laser beam was split off by a beam splitter (BS) and the laser energy was measured by a power detector. The number of laser shots applied to the sample was controlled by an electromechanical shutter. The laser beam was tightly focused onto the fiber by a 10x objective lens (numerical aperture 0.26, M Plan Apo NIR, Mitutoyo) mounted on a Z stage. The multi-D-shaped trench under fabrication was translated by a computer controlled X-Y micro-positioning stage with error less than 1 μm. The fabrication process was monitored *in situ* by a charge-coupled device (CCD).

**Figure 3.**
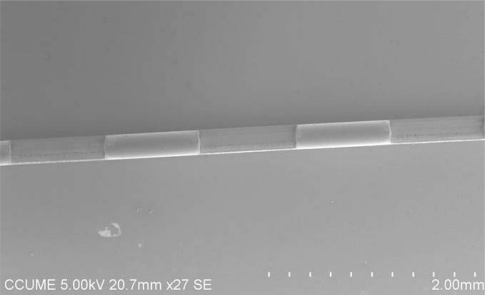
SEM image of a multi-D-shaped optical fiber.

**Figure 4.**
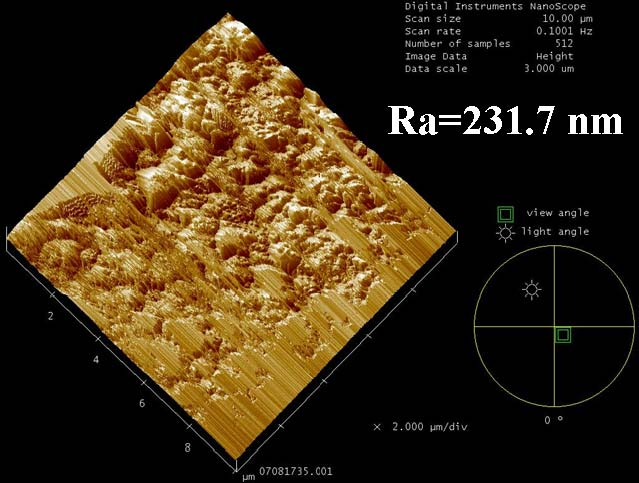
AFM image of the exposed core surface (scanning area 10 μm × 10 μm).

Shown in Figures 1 and 3 are the representative diagram and the SEM image of the fabricated multi-D-shaped optical fibers, respectively. The diagram shows that the depth of a single D-shaped zone was 100 μm and the length was 1 mm. The space between the core center and the flat surface of the fiber was 25 μm. It indicates that the core of the fiber has been exposed and part of the jacket layer has provided enough mechanical strength for further processing. Since the material properties of the jacket layer and cladding layer are much different, there are three steps applied for fabricating the multi-D-shaped optical fibers. In Step 1, the jacket layer mainly composing of polymer was removed with a relatively high energy of 20 mW, and the scanning width and length were set to 100 μm and 1 mm, respectively. After processing of Step 1, part of jacket was exposed. In Step 2 the femtosecond laser focus spot was then varied in height up to the cladding surface. The processing parameters were the same as Step 1 and the procedure kept on repeat until the material of D-shaped zone was all removed. A surface treatment was carried out in Step 3. In this process, a relatively high scanning speed (0.3 mm/s) and defocus strategy (30 μm above bottom of the D-shaped zone) for the annealing treatment were applied. As shown in Figure 4, the surface mean roughness is 231.7 nm as measured by an AFM (model Multi Mode, Veeco, Inc.). Such surface quality allows the light easily propagating inside the D-shaped fiber to interact with surrounding medium through the evanescent wave or light reflection.

**Figure 5.**
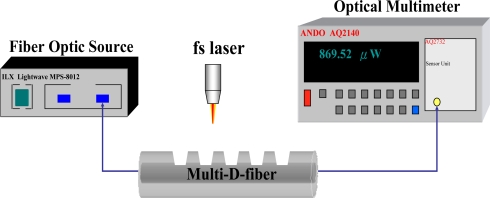
Experimental setup for monitoring the transmission power loss of multi-D-shaped optical fibers during fabrication process.

During the fabrication of the multi-D-shaped optical fiber, the transmission power was monitored with a fiber optic light source (λ = 1,550 nm, model MPS-8012, Lightwave, Inc.) and a multifunction optical meter (model AQ2140, ANDO, Inc.). The experimental setup is shown in Figure 5. For the multi-D-shaped fiber with five D-shaped zones, the average power loss measured for four samples was 0.92 ± 0.31 dB (dB loss = 10 × logP_2_/P_1_, P_1_ = input power, P_2_ = output power) It can be seen that multi-D-shaped optical fiber produces about 22 ± 4% transmission power loss while keeping enough transmission power for further testing.

**Figure 6.**
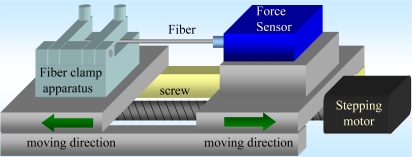
Schematic diagram of the tensile test machine.

The tensile tests of the fibers after manufacturing were performed. Tensile testing is a standard procedure for determining the mechanical properties of materials. A standard tension test machine is shown in Figure 6. The multi-D-shaped optical fiber was placed and fixed in the grips of the testing machine. The grips were driven by stepping the motor (the minimum displacement is 1 μm) as well as the screw, hence the load applied by the machine was axial. The testing machine elongated the multi-D-shaped optical fiber at constant rate until the grooved optical fiber ruptured. During the test, continuous readings of the applied load and the elongation of the multi-D-shaped optical fiber were taken. The load-elongation curve diagrams for the optical fibers could be obtained to measure their mechanical strengths.

**Figure 7.**
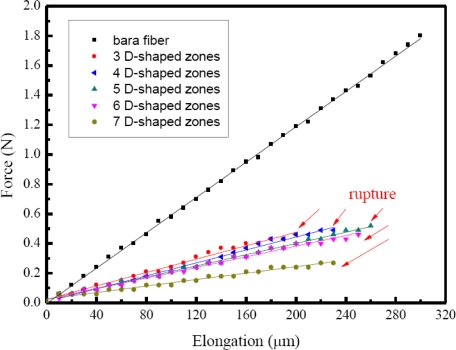
The tensile testing curve diagrams for a bare optical fiber and five different D-shaped fibers.

Figure 7 depicts the load-elongation curve diagrams for a bare fiber and five different D-shaped fibers. The symbols representing the experimental data and linear least square fitting of these data are also plotted. Fitting curves (straight line type) for five different D-shaped fibers are close to that of the bare fiber without any processing. However, the multi-D-shaped optical fiber ruptured when its elongation was beyond 250 μm, which was only two-three of that for a bare fiber. The force constant of a bare fiber was determined to be 5,956 N/m, while for those of multi-D-shaped fibers force constants were determined to be from 1,089 N/m to 2,256 N/m. It is shown that multi-D-shaped fibers have sufficient mechanical strength to resist damage from handling and packaging.

## 3. Refractive Index Measurements

**Figure 8.**
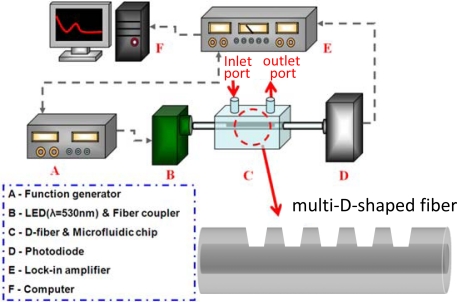
Schematic of the experimental setup for refractive index measurements with the multi-D-shaped optical fiber. The inlet and outlet ports of the microfluidic chip are used to infuse sucrose solution flowing through the sensing fiber.

Figure 8 is an illustration of the experimental setup for refractive index sensing measurements with the multi-D-shaped optical fiber sensor. The fiber-optic sensing system used to measure the transmission power of the sensor was consisted of a function generator (model GFG-8255A, Good Will Instrument, Inc.), a light emission diode (LED) light source (model EHP-AX08LS-HA/SUG01-P01, Everlight Electronics Co., Ltd), a sensing multi-D-shaped fiber, a microfluidic chip, a photodiode (model 2001, New Focus, Inc.), a lock-in amplifier (model 7225R, EG&G Instrument, Inc.) and a computer for data acquisition.

The interrogation of the sensor based on intensity modulation was performed by launching a LED light source propagating through the sensing multi-D-shaped fiber into a photodiode. The LED as an excitation light source was modulated by a function generator with a square wave current at a frequency of 1 kHz and a voltage of 3.5 V. Through a fiber collimator (model F240FC-A, Thorlab) light with a wavelength of 530 nm emitted from the LED was coupled into the optical fiber and was carried through the sensing portion of the D-shaped fiber which was immersed in different concentrations of sucrose solution. For measurements of transmission power, the ATR signals emitted from the sensing fiber was measured by a photodiode and the light signal was converted into electronic signal in voltage. A lock-in amplifier operating at 1 kHz chopping frequency was used to for phase-shift detection of the photodiode output signal and for increasing the signal to noise ratio. With the lock-in, low level optical signals can be detected with full-scale voltage sensitivities down to 2 nV and dynamic reserve exceeding 100 dB. The output from the lock-in-amplifier was recorded at a sampling rate of 1 Hz using a computer with a data acquisition system. For each run of transmission power measurement, the average transmission power can thus be obtained by evaluating a series of 500 data samples. In this scheme the computer associated with a photodiode and a lock-in amplifier carried out the light intensity demodulation and signal processing for obtaining the output signal of the sensor.

**Figure 9.**
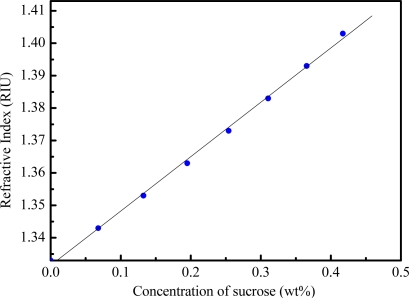
Refractive index of the sucrose solution at different concentrations.

The ability of the multi-D-shaped optical fibers to detect changes in the surrounding refractive index was studied. The control of surrounding refractive index was through the use of sucrose solutions with various concentrations [10]. The relationship between refractive index and concentration for sucrose solution in the range of 1.333 to 1.403 is shown in Figure 9. The transmitted ATR signal of the multi-D-shaped fiber excited by the LED light source changed linearly with an increase of the surrounding refractive index. Different concentrations of sucrose solution were used to measure the sensitivity of the multi-D-shaped fiber sensor. The fiber sensor was immersed in sucrose solutions and its transmission power or intensity was measured with the photodiode and lock-in-amplifier. Because of the better linearity of the plot of transmission power or intensity *versus* refractive index, transmission power or intensity was used as the sensor response in this study. For precise refractive index measurement, we kept the experimental setup and sample solution at a constant ambient temperature (within 0.1 °C fluctuation). The sensing fiber was placed inside the microfluidic chip and a small fixed magnitude of tension was applied to minimize bending of the fiber. Therefore, the results reported here were not influenced by temperature, strain and bending effects.

**Figure 10.**
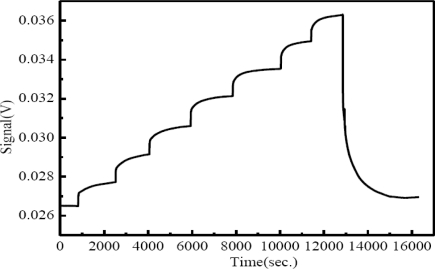
Plot of temporal response of the fiber sensor with respect to injection of an increasing sequence of concentrations or refractive indices of sucrose.

When the concentration and, hence, the refractive index of a sucrose solution increased in the range of 1.333–1.403, the transmitted light intensity of the fiber sensor exhibited a linear increase in the output power. Figure 10 shows the time course of sensor response as a slowly increasing sequence of concentrations of sucrose was injected into the sensing microfluidic chip. After the final injection of sucrose solution, the sensing microfluidic chip was injected with water. It can be seen that the sucrose solution could be eluted completely and quickly, and the sensor response was back to the original intensity level. The sensor response was reversible because such injection and elution processes were repeatable.

**Figure 11.**
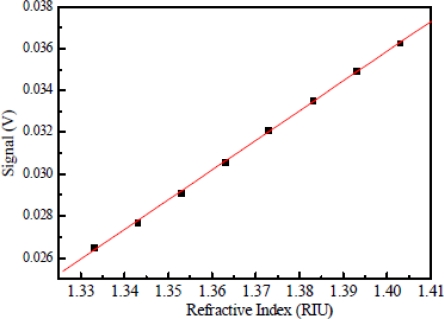
Plot of sensor response *versus* refractive index of the sucrose solution for a sensing fiber with five D-shaped zones.

**Figure 12.**
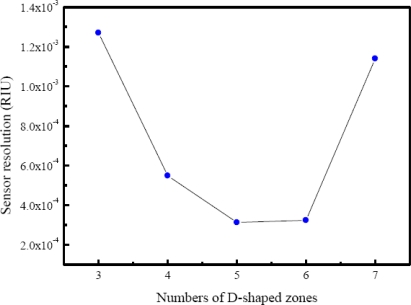
Plot of sensor response *versus* the number of D-shaped zones.

A linear regression method was employed to analyze the relationship between sensor response and refractive index changes. This method calculated the best-fitting linear equation (straight line) for the observed data using the least squares approach. The slope of the linear fit to the measured sensor response at various surrounding refractive index changes was determined as the refractive index sensitivity of the investigated fiber sensor. Figure 11 shows a linear fit (correlation coefficient *R* = 0.9987) to the plot of sensor response as a function of the refractive index for a sensing fiber with five D-shaped zones. Table 1 lists standard deviations of sensor response at various concentrations, which were quite stable during the testing. Results for a sensing fiber with five D-shaped zones show that the refractive index sensitivity of the fiber sensor is 0.142 V/refractive index unit (RIU), which leads to a refractive index resolution (sensor resolution = 3*σ*/*m*, *σ* = standard deviation of sensor response in measuring the blank sample, *m* = slope) of 3.13 × 10^−4^ RIU in index. We have collected and analyzed the data by varying the number of D-shaped zones in the sensing fiber. Figure 12 shows a plot of sensor resolution *versus* the number of D-shaped zones. When the number of D-shaped zones was changed from three to seven, we found that the sensor resolution increased with the number of D-shaped zones and decreased after showing a maximum value at five D-shaped zones. Studies presented here demonstrate that these multi-D-shaped fiber sensors can provide a resolution of 1.27 × 10^−3^–3.13 × 10^−4^ for refractive indices in the range of 1.333 to 1.403.

Compared with other existing index sensing schemes, such sensor performance is more promising and favorable. For example, Abbe refractometers have a resolution of 1 × 10^−4^–2 × 10^−5^ for indices 1.33 to 1.58 and are relatively bulky and expensive. Sensors based on FBG evanescent wave couplings yield an index resolution of only around 1 × 10^−3^ and require a more complex optical layout. Long-period fiber grating (LPFG) sensors based on resonant wavelength shifts provide an index resolution of 1 × 10^−3^–2 × 10^−4^ [11,12] and require some expensive and high precision instruments such as laser source and optical spectrum analyzer. Our fiber sensor is low-cost and compact, has comparable or better resolution, and can be used remotely. The results reported here demonstrate that the multi-D-shaped fibers are attractive for chemical, biological, and biochemical sensing with aqueous solutions.

## 4. Conclusions

Studies presented here successfully demonstrate the feasibility of fabricating a class of high sensitive refractive-index sensor based on the multi-D-shaped optical fiber written by femtosecond laser pulses. The multi-D-shaped fibers were fabricated using multimode standard communications step-index optical fiber with a core diameter of 62.5 μm and with an outer cladding diameter of 125 μm. The realization of the sensor is through the measurement of transmitted light intensity of the sensing fiber. When exposing the D-shaped fiber to sucrose solutions of increasing refractive index, the sensor response increases linearly. By transmission power interrogation, we demonstrate that the multi-D-shaped fiber sensors can provide a limiting resolution of 1.27 × 10^−3^–3.13 × 10^−4^ RIU for refractive indices in the range of 1.333 to 1.403. Such a highly sensitive fiber-optic refractive index sensor is suitable for use as a chemical or biological sensor. The advantage of this type of sensor is relatively simple of construction, compact, low cost, and ease of use. Moreover, the sensor has the potential capability for on-site, *in vivo*, and remote sensing, and has the potential use for disposable sensors.

## Figures and Tables

**Table 1.** Standard deviation of sensor response at different refractive indexes for a sensing fiber with five D-shaped zones.

**Refractive index, (RIU)**	**Slope, m (V/RIU)**	**Standard deviation, σ (V)**
1.333		7.15 × 10^−6^
1.343		2.22 × 10^−5^
1.353		2.56 × 10^−5^
1.363	0.142	1.45 × 10^−5^
1.373		9.3 × 10^−6^
1.383		5.1 × 10^−6^
1.393		1.71 × 10^−5^
1.403		1.76 × 10^−5^
